# Visual art experience and psychological distress among university students: parallel indirect effects through presence and search for meaning

**DOI:** 10.3389/fpubh.2026.1927579

**Published:** 2026-09-03

**Authors:** Huanqin Lv

**Affiliations:** College of Arts and Design, Ningbo University of Finance and Economics, Ningbo, Zhejiang, China

**Keywords:** health promotion, meaning in life, psychological distress, public health education, university students, visual art experience

## Abstract

**Purpose:**

University students often experience academic pressure, interpersonal challenges, and uncertainty about future development, making psychological distress an important concern for health promotion in higher education. Visual art experience may provide opportunities for emotional expression, aesthetic immersion, self-reflection, and meaning-making. However, its association with psychological distress and the possible statistical indirect roles of presence of meaning and search for meaning remain insufficiently examined. This study therefore examined these associations in a cross-sectional university-student sample.

**Methods:**

A cross-sectional online questionnaire survey was conducted within a single institutional context using non-probability online recruitment beginning on April 22, 2026. The analytic archive contained 500 submitted questionnaire records, of which 423 were retained after data-quality screening and 77 were excluded. Participants completed measures of visual art experience, presence of meaning, search for meaning, depressive symptoms, anxiety symptoms, and stress symptoms. Analyses included reliability and psychometric evaluation of the study-specific measures, Pearson correlations, hierarchical regression with assumption diagnostics, domain-specific outcome analyses, exploratory effect-modification tests, and a 5,000-resample bootstrap analysis of parallel indirect effects. Because no probability-sampling frame or invitation/start denominator was available, population representativeness and a conventional survey response rate are not claimed.

**Results:**

Visual art experience was negatively associated with psychological distress (*r* = −0.37, *p* < 0.001) and positively associated with both presence of meaning (*r* = 0.62, *p* < 0.001) and search for meaning (*r* = 0.12, *p* < 0.05). Presence of meaning was negatively associated with psychological distress (*r* = −0.43, *p* < 0.001), whereas search for meaning was not significantly associated with psychological distress. In the primary parallel indirect-effect analysis, the indirect effect through presence of meaning was significant (95% bootstrap CI [−8.988, −4.151]), whereas the indirect effect through search for meaning was not significant (95% bootstrap CI [−0.490, 0.327]).

**Conclusion:**

These findings provide preliminary cross-sectional evidence that visual art experience is associated with lower psychological distress among university students. The pattern of results is statistically consistent with an indirect pathway through presence of meaning, but it does not establish a causal psychological mechanism. Presence of meaning and search for meaning should be interpreted as distinct dimensions, and the findings should be understood as associations rather than causal effects.

## 1 Introduction

In recent years, the mental health of university students has become an increasingly important issue in higher education and public health promotion. Universities are not only educational institutions but also important settings for early prevention, health education, and mental health promotion among young adults. University life represents a critical transitional period from late adolescence to early adulthood, during which students must cope with academic pressure, uncertainty about future employment, interpersonal changes, and the development of self-identity. Against this background, psychological distress often manifests as depressive symptoms, anxiety symptoms, and stress, which may further affect students’ academic engagement, social adjustment, and psychological well-being (1–3). Psychological distress among university students is multidimensional and heterogeneous and should therefore be considered in relation to individual psychological resources, social support, and meaning-related factors (4). Within this context, identifying accessible, non-clinical, and education-based resources for psychological support is of both theoretical and practical importance.

Evidence linking the arts with mental health spans heterogeneous forms of engagement, including formal arts education, active artistic creation, receptive appreciation, and immersive exposure. These forms should not be treated as interchangeable because they differ in intensity, agency, setting, and psychological demands. Likewise, previous studies have examined outcomes ranging from well-being, mood, creativity, and self-efficacy to stress tolerance, anxiety, and depressive symptoms. Positive well-being outcomes are conceptually related to, but not equivalent to, psychological distress. The present study therefore focuses specifically on self-reported visual art experience and its association with depressive, anxiety, and stress symptoms.

In the present study, visual art experience is conceptualized broadly as students’ self-reported engagement with and psychological responses to visual artworks. It includes content relating to visual appreciation, creative participation, emotional resonance, aesthetic immersion, and self-reflection. These five areas are treated as content domains used to organize the study-specific items, rather than as established latent dimensions of a previously validated scale. This distinction is important because the construct is broader than any single form of art education, art creation, museum exposure, or art therapy.

Prior findings suggest that different art-related activities may be associated with different psychological outcomes. Formal fine-arts and aesthetic-education studies have primarily examined psychological well-being and educational resources such as creativity, self-efficacy, and basic psychological needs (5, 6). Research on immersive art exposure has focused on short-term mood and stress responses (7), whereas drawing practice has been examined in relation to resilience, self-disclosure, and distress tolerance (8). Collectively, this literature supports the relevance of artistic engagement to psychological functioning, but it does not establish that these heterogeneous activities are equivalent to the broad self-reported construct of visual art experience used here. It also leaves open the more specific question of whether visual art experience is associated with depressive symptoms, anxiety symptoms, stress, and overall psychological distress among university students.

Meaning in life provides a theoretically relevant candidate pathway because it concerns the extent to which individuals experience purpose, coherence, and direction. The Meaning in Life Questionnaire distinguishes presence of meaning from search for meaning (9). Presence reflects an existing sense that life is meaningful, whereas search reflects active efforts to find or deepen meaning. These dimensions are theoretically distinct and may relate differently to psychological distress; they are therefore analyzed separately rather than assumed to form a single homogeneous mediator.

Visual art experience may be associated with meaning in life because viewing or creating artworks can involve symbolic interpretation, emotional expression, self-reflection, and engagement with themes of identity, memory, relationships, culture, and life goals (10, 11). These processes provide a conceptual basis for testing whether presence of meaning and search for meaning statistically account for part of the association between visual art experience and psychological distress. Because the study is cross-sectional, any such pattern is interpreted as a statistical indirect effect rather than evidence of temporal or causal mediation.

Three specific gaps motivate the study. First, much of the literature concerns formal arts education, discrete artistic activities, or positive well-being outcomes rather than a broad self-reported visual art experience construct and psychological distress. Second, presence of meaning and search for meaning are often discussed under the general label of meaning in life even though their psychological functions may differ. Third, evidence is limited on whether these two dimensions show different indirect associations between visual art experience and psychological distress in university students. The present study addresses these gaps using separate hypotheses and a parallel indirect-effect model.

Based on this theoretical framework, the hypotheses are formulated separately for presence of meaning and search for meaning:

*H1*: Visual art experience is negatively associated with psychological distress among university students.

*H2a*: Visual art experience is positively associated with presence of meaning among university students.

*H2b*: Visual art experience is positively associated with search for meaning among university students.

*H3a*: Presence of meaning is negatively associated with psychological distress among university students.

*H3b*: Search for meaning is negatively associated with psychological distress among university students.

*H4a*: The association between visual art experience and psychological distress shows a statistically significant indirect effect through presence of meaning.

*H4b*: The association between visual art experience and psychological distress shows a statistically significant indirect effect through search for meaning.

**Figure 1:**
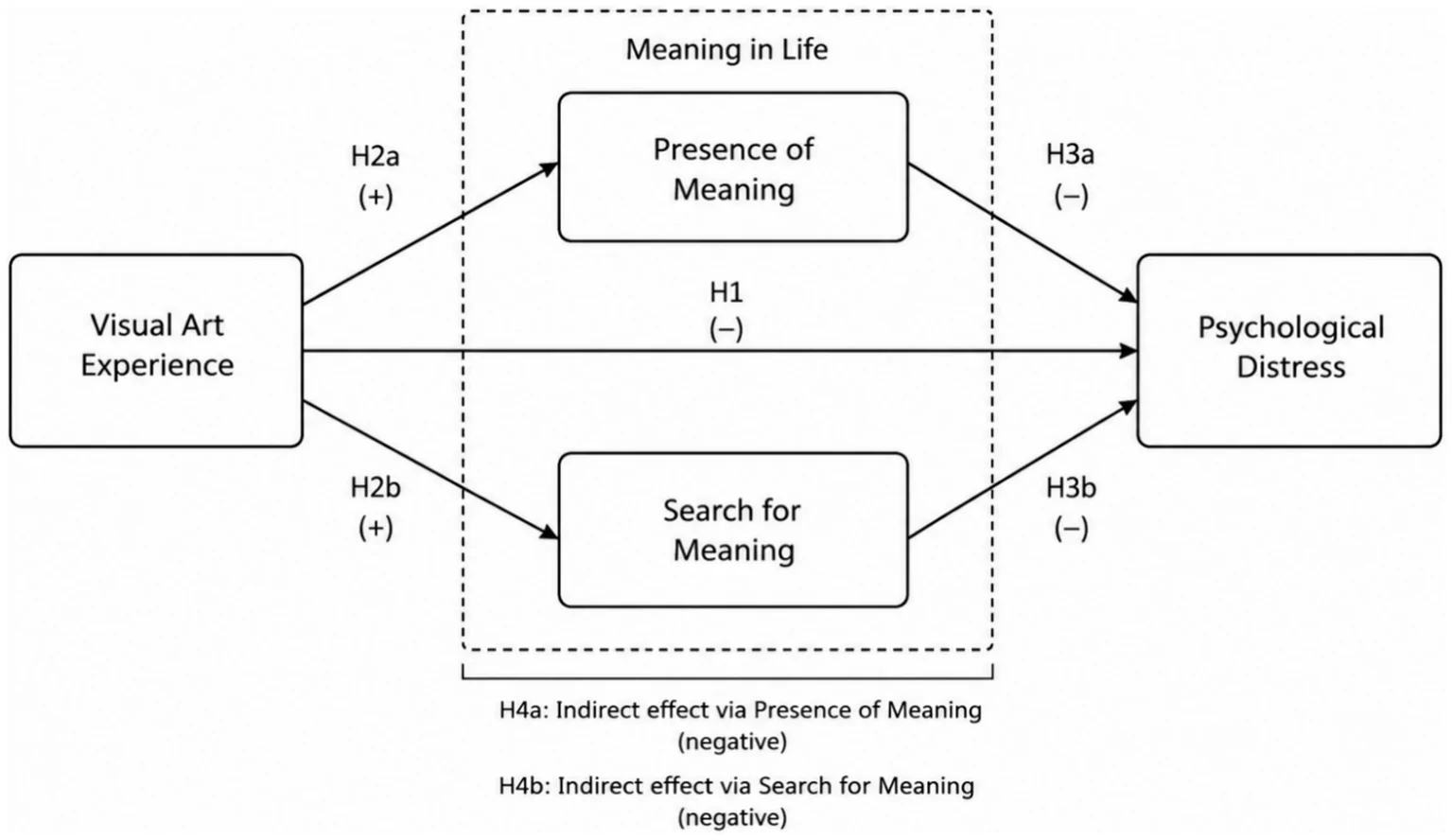
Revised hypothesized model distinguishing presence of meaning and search for meaning. (+) indicates a positive association; (−) indicates a negative association. H4a and H4b are tested as parallel indirect effects.

The hypothesized model treats presence of meaning and search for meaning as parallel, theoretically distinct variables. Figure 1 summarizes the proposed associations; the indirect-effect hypotheses are tested statistically and are not interpreted as causal mechanisms.

In summary, the study examines whether a broad self-reported visual art experience construct is associated with psychological distress and whether the observed association is statistically consistent with distinct indirect paths through presence of meaning and search for meaning. This framing differentiates the present construct from specific forms of arts education or art intervention and keeps interpretation within the limits of a cross-sectional design.

## 2 Literature review

### 2.1 Visual art experience and mental health

Visual art engagement can involve receptive activities such as viewing paintings, photographs, design, sculpture, video, and digital art, as well as active creation. Prior evidence generally suggests associations between arts engagement and health-related outcomes, but the literature spans different populations, settings, activity types, and outcome measures (12, 13). Accordingly, findings from formal art education, active creation, passive appreciation, or immersive environments should be interpreted as related but not interchangeable evidence (21).

At a broader level, review and population-based evidence indicates that arts engagement may be associated with health-related outcomes through emotional, cognitive, social, and meaning-related processes, while receptive and participatory forms of engagement may differ in their psychological correlates (12–14). This literature supports the plausibility of an association between art-related experience and mental health, but its heterogeneity also cautions against treating formal education, active creation, passive appreciation, immersive exposure, and broad self-reported experience as equivalent. The present study narrows this literature by focusing on psychological distress-depressive symptoms, anxiety symptoms, and stress-rather than assuming that findings for positive well-being automatically generalize to distress outcomes (22, 23).

### 2.2 Psychological distress in university health promotion

Psychological distress refers here to self-reported depressive symptoms, anxiety symptoms, and stress symptoms rather than to a clinical diagnosis. University students may experience these symptoms in relation to academic demands, interpersonal difficulties, financial or employment uncertainty, and developmental transitions. Because distress is not simply the inverse of positive well-being, evidence that an activity improves well-being cannot automatically be interpreted as evidence that it reduces depression, anxiety, or stress.

From a health-promotion perspective, non-clinical educational experiences may complement, but cannot replace, professional psychological services. The present study therefore examines visual art experience as a potential educational correlate of psychological distress rather than as a treatment or therapeutic intervention.

### 2.3 Meaning in life as a psychological resource in health promotion

Meaning in life is commonly conceptualized as the subjective experience that one’s life has purpose, coherence, and significance. The MLQ distinguishes presence of meaning from search for meaning (9). Presence generally reflects an existing sense of purpose and coherence, whereas search reflects active exploration. Treating them separately is theoretically important because searching for meaning may occur either as constructive exploration or in the context of uncertainty.

Prior research links presence of meaning more consistently with psychological adjustment and lower distress, while the correlates of search for meaning are more context-dependent (15, 16). Research among university students similarly indicates that meaning-related resources are connected with self-acceptance, social support, and mental-health profiles (4, 17). These findings support testing the two MLQ dimensions separately.

### 2.4 The relationship between visual art experience and meaning in life

Visual art experience may be associated with meaning-related processes because artworks often contain symbolic, narrative, cultural, and existential content. Viewing or creating art can invite interpretation of emotions, identity, relationships, memory, values, and future goals. Aesthetic experience is therefore not merely visual exposure; it can include emotional, perceptual, cultural, understanding-related, and flow-related processes (10).

This theoretical connection does not imply that all art activities produce meaning or that meaning necessarily follows art exposure. Rather, it provides a rationale for testing whether students who report richer visual art experiences also report stronger presence of meaning or greater search for meaning. The two associations are examined separately because the MLQ dimensions are conceptually distinct.

### 2.5 Meaning in life as a candidate indirect pathway

The proposed model tests whether presence of meaning and search for meaning statistically account for part of the association between visual art experience and psychological distress. This proposal is consistent with broader evidence that positive experiences and psychological resources can be linked with meaning-related processes and adjustment (17, 18). However, because the current data are cross-sectional, the model cannot establish temporal ordering or causal mediation.

The parallel model is the primary analysis. Presence of meaning is expected to show a stronger inverse association with psychological distress because it reflects an established sense of purpose and coherence. Search for meaning may show a weaker or different pattern because active searching can coexist with either personal growth or unresolved uncertainty. Testing these paths separately avoids obscuring theoretically meaningful differences in a single total meaning-in-life score.

## 3 Methods

### 3.1 Research design and data source

This study adopted a cross-sectional questionnaire design to examine the associations among visual art experience, presence of meaning, search for meaning, and psychological distress among university students. The study further examined whether the association between visual art experience and psychological distress was statistically consistent with parallel indirect effects through presence of meaning and search for meaning. Because all study variables were measured at a single time point, the analyses were intended to estimate associations and statistical indirect effects rather than temporal or causal relationships.

Data were collected through an online questionnaire coordinated within the institutional context of Ningbo University of Finance and Economics. Recruitment began on April 22, 2026, using a non-probability online recruitment approach. Because the study did not use a probability-based sampling frame, the sample should not be interpreted as representative of the wider university-student population. Eligible participants were university students aged 18–25 years who voluntarily agreed to participate and submitted the questionnaire. Participants received an incentive for participation, with the provision of incentives documented in the project records dated May 20, 2026. The retained survey export contains 500 submitted questionnaire records. Because the number of students who received or opened the survey link was not retained, a conventional survey response rate cannot be calculated.

Before inferential analysis, the 500 submitted questionnaire records were screened for data quality. The archived screening fields can be reproduced by four operational rules: (1) an attention-check response other than the expected response of 4; (2) completion time shorter than 180 s; (3) five or more missing item responses; and (4) an obvious straight-line pattern in which the same response option was selected throughout each major multi-item questionnaire block. These rules identified 24 attention-check failures, 20 unusually short completions, 15 records with excessive missing data, and 18 straight-line records, respectively, for a total of 77 exclusions. Each excluded record has one primary exclusion reason in the archive, and no overlap is observed among the four reconstructed screening flags; therefore, no case is double-counted. The screening rules were applied before the reported statistical analyses, but the retained files do not contain a dated preregistration or protocol that would allow us to verify that each numerical threshold had been formally specified *a priori* before data collection. After screening, 423 records were retained, corresponding to an analytic retention proportion of 84.60%, not a survey response rate.

This questionnaire study involved human participants. The study protocol was reviewed and approved by the Research Ethics Committee of the School of Art and Design, Ningbo University of Finance and Economics (Approval No. NBUFE-SAD-REC-2026-002; approved on April 20, 2026) before recruitment began. Data collection commenced on April 22, 2026. Participation was voluntary, and informed consent was obtained from all participants before completion of the questionnaire. Participants were informed of the study purpose, questionnaire content, anonymous nature of the survey, intended use of the data, and their right to decline participation or withdraw without penalty. No names, student identification numbers, contact details, or other directly identifying information were collected.

### 3.2 Measures and variable measurement

All questionnaire items were administered in Chinese. The visual art experience items were newly worded for this study on the basis of literature on aesthetic experience, arts engagement, emotional response, immersion, and reflection rather than copied verbatim from a single validated instrument. The 10 meaning-in-life items were adapted with conceptual reference to the Meaning in Life Questionnaire (MLQ; (9)) and Chinese MLQ validation literature (19), while the 21 psychological-distress items were adapted with reference to the DASS-21 framework (1, 2) and Simplified Chinese DASS-21 validation literature (20). The administered MLQ- and DASS-referenced items were not verbatim copies of the published Chinese instruments. The retained project materials do not document a separate forward-translation/back-translation procedure for these study-specific adaptations; therefore, the revised manuscript does not claim psychometric equivalence with the published C-MLQ or Simplified Chinese DASS-21. Exact Chinese and English item wording is provided in Supplementary material.

#### 3.2.1 Demographic information

Demographic information was collected to describe the characteristics of the sample and to provide covariates for subsequent analyses. The demographic variables included gender, age, grade, major type, frequency of visual art exposure, and participation in art-related courses or activities. Major type, frequency of visual art exposure, and participation in art-related activities were included as covariates where appropriate because students with different academic backgrounds and levels of art engagement may differ systematically in their exposure to and experience of visual art.

#### 3.2.2 Visual art experience

Visual art experience was measured with a study-specific 15-item instrument developed for the present study. The items were newly written, literature-informed statements intended to represent a broad self-reported experience of visual art, including appreciation, active engagement/creation, emotional resonance, immersion, and self-reflection/meaning-making. Before formal administration, the wording was qualitatively reviewed by researchers with art- and psychology-related expertise for clarity and content relevance. The retained archive contains the final 15 items but does not preserve the initial item pool, item-deletion history, the number or identities of expert reviewers, or item-level content-validity index ratings; therefore, no numerical content-validity coefficient is claimed. The five areas are treated as conceptual content-organizing domains rather than as five empirically established subscales. The complete retained Chinese item wording and English renderings are provided in Supplementary Table S1.

The participant-level analytic file contains observed responses from 1 through 7 for every visual art experience item, and the archived VAE_Total variable is exactly the mean of the 15 recorded item responses. Accordingly, the revised scoring description uses a 7-point Likert range from 1 = strongly disagree to 7 = strongly agree, and the mean of the 15 items is used as the overall visual art experience score. The five conceptual content areas are not scored as separate subscales. Internal consistency in the analytic sample was high (Cronbach’s alpha = 0.902). To evaluate whether use of a single overall score was empirically defensible, the 423 retained cases were divided reproducibly into an exploratory subsample (*n* = 211) and a confirmatory subsample (*n* = 212). Parallel analysis and principal-axis EFA supported one dominant factor, and the one-factor CFA showed good fit in the confirmatory subsample. Composite reliability was strong, although AVE remained below 0.50; therefore, the total score is treated as a preliminary predominantly unidimensional index rather than as a fully validated instrument.

#### 3.2.3 Meaning in life

Meaning in life was assessed using a study-specific 10-item Chinese adapted measure organized into two theoretically distinct five-item dimensions: presence of meaning and search for meaning. Item content was developed with reference to the original MLQ framework (9) and the Chinese MLQ literature (19), but the administered items were adapted and were not a word-for-word copy of the published C-MLQ. Because no independent translation/back-translation record was retained, the present item set is described as MLQ-referenced rather than as the validated Chinese MLQ itself. Presence of meaning was represented by five positively keyed items concerning an existing sense of meaning, valued goals, life direction, and meaning in daily experience; search for meaning was represented by five positively keyed items concerning active exploration of meaningful goals, values, and life priorities. The exact Chinese and English item wording is provided in Supplementary Table S3.

All 10 adapted meaning-in-life items were rated on a 7-point scale from 1 = absolutely untrue to 7 = absolutely true. Mean scores were calculated separately for presence of meaning and search for meaning; no overall meaning-in-life score was used in the primary analysis because the two dimensions are conceptually distinct. No reverse scoring was required for the adapted item set. Cronbach’s alpha was 0.780 for presence of meaning and 0.754 for search for meaning. In the present sample, a correlated two-factor CFA fit well and substantially outperformed a one-factor alternative, supporting empirical separation of presence and search within this dataset. However, AVE values were below 0.50, so these analyses are interpreted as internal structural evidence rather than independent validation of the adapted Chinese items.

#### 3.2.4 Psychological distress

Psychological distress was assessed using a study-specific 21-item Chinese adapted measure organized into three seven-item symptom domains: depressive symptoms (PD01-PD07), anxiety symptoms (PD08-PD14), and stress symptoms (PD15-PD21). The three-domain organization was developed with reference to the depression-anxiety-stress framework of the DASS-21 (1, 2) and relevant Chinese validation literature (20), but the administered items were adapted Chinese-language items rather than the verbatim validated Simplified Chinese DASS-21. Importantly, the archived questionnaire-item file confirms that every psychological-distress item explicitly instructed participants to report their experience during the past week. Responses were recorded from 0 = did not apply/not present to 3 = very marked. The exact adapted item wording is provided in Supplementary Table S4.

For each adapted symptom domain, the seven item scores were summed to produce a raw score from 0 to 21. The archived analysis file confirms that each raw domain score was multiplied by two, yielding transformed depression, anxiety, and stress scores from 0 to 42. Overall psychological distress was calculated as the sum of the three transformed domain scores (possible range 0–126). The multiplication by two is a linear rescaling retained from the DASS-21 scoring convention that informed the analysis; it does not imply that these adapted items constitute a verbatim validated DASS-21 administration. DASS-21 diagnostic or severity cutoffs were not applied. In response to the reviewer’s concern about reliance on a combined outcome, the revised analysis reports depressive symptoms, anxiety symptoms, and stress symptoms separately as co-outcomes alongside the overall psychological-distress summary score.

In the present sample, Cronbach’s alpha was 0.850 for the adapted depressive-symptom items, 0.822 for the anxiety-symptom items, 0.840 for the stress-symptom items, and 0.912 across all 21 psychological-distress items. The measure is interpreted as a study-specific self-report operationalization of depressive, anxiety, and stress symptoms rather than as a clinical diagnostic instrument or a verbatim DASS-21 administration. Additional internal structural analyses of the intended three-domain organization are reported in Section 4.2 (see Table 1).

**Table 1:** Measurement and coding of the main variables.

Variable type	Variable	Meaning	Measurement	Scoring
Independent variable	Visual art experience	Broad self-reported engagement with and psychological responses to visual art; conceptual content areas include appreciation, creation/engagement, emotional resonance, immersion, and self-reflection	Study-specific 15-item visual art experience instrument	7-point Likert scale; overall mean score; content areas not scored as separate subscales
Mediating variable	Presence of meaning	Existing sense that life is meaningful, purposeful, and coherent	Adapted 5-item Presence-of-Meaning item set	7-point scale (1–7); all items positively keyed; mean score calculated separately
Mediating variable	Search for meaning	Active efforts to find or deepen life meaning	Adapted 5-item Search-for-Meaning item set	7-point scale (1–7); all items positively keyed; mean score calculated separately
Dependent variable	Psychological distress	Self-reported depressive, anxiety, and stress symptoms during the past week	Adapted 21-item psychological-distress measure (7 depression, 7 anxiety, 7 stress items)	Items scored 0–3; raw domain scores 0–21; archived analysis scores multiplied by two to 0–42; overall psychological distress 0–126; no DASS-21 clinical cutoffs applied
Control variables	Gender, age, grade, major type, art exposure frequency, art-related activity participation	Basic sample characteristics	Demographic items	Coded as categorical or continuous variables

### 3.3 Data processing and statistical analysis

Before statistical analysis, all 500 submitted records were screened using the four operational rules reconstructed from the archived screening fields: attention-check response ≠ 4 (*n* = 24), completion time <180 s (*n* = 20), at least five missing item responses (*n* = 15), and a complete straight-line response pattern across the major questionnaire blocks (*n* = 18). The categories are mutually exclusive in the archived screening file and sum to 77 excluded records, leaving 423 for analysis. The retained project records do not contain invitation/start denominators or a dated preregistration documenting that these thresholds were specified before data collection; therefore, the 84.60% figure is reported only as an analytic retention proportion. Descriptive statistics were subsequently calculated for demographic characteristics and the main study variables.

Reliability analysis focused on internal consistency within conceptually coherent measures. Cronbach’s alpha was reported for the visual art experience instrument, the two adapted meaning-in-life dimensions, and the adapted psychological-distress domains. Because the Visual Art Experience Scale was newly developed, the 423 retained cases were additionally divided by a reproducible random split (seed = 20,260,810) into an exploratory subsample (*n* = 211) and a confirmatory subsample (*n* = 212). In the exploratory subsample, Kaiser-Meyer-Olkin (KMO) and Bartlett’s tests were used as factorability checks, parallel analysis was used to determine the number of factors, and principal-axis factoring was used to estimate EFA loadings. The factor structure suggested by EFA was then tested by CFA in the independent confirmatory subsample. Model fit was evaluated using Chi-square, CFI, TLI, RMSEA, and SRMR. Composite reliability and AVE were calculated as indicators relevant to convergent validity, and HTMT values between visual art experience and the other study dimensions were examined as evidence of discriminant validity. KMO and Bartlett statistics were not interpreted as standalone evidence of construct validity. Harman’s single-factor test was retained only as a preliminary diagnostic of common method variance.

Because the meaning-in-life and psychological-distress items were adapted rather than administered as verbatim validated Chinese scale versions, additional full-sample CFAs were conducted as supplementary internal structural checks. The 10 adapted meaning-in-life items were tested as a correlated two-factor model (presence of meaning and search for meaning), and the 21 adapted psychological-distress items were tested as a correlated three-factor model (depressive, anxiety, and stress symptoms); one-factor alternatives were fitted for comparison. Treating the item scores as approximately continuous, models were estimated by normal-theory maximum likelihood using Pearson item correlation matrices. Fit was summarized with chi-square, CFI, TLI, RMSEA, and SRMR, and composite reliability (CR), average variance extracted (AVE), and HTMT were calculated. Because these analyses are *post hoc*, use the same analytic sample, and include ordinal items, they are interpreted as internal psychometric evidence rather than independent validation of the adapted measures.

Pearson correlations were used to examine bivariate associations among visual art experience, presence of meaning, search for meaning, depressive symptoms, anxiety symptoms, stress symptoms, and overall psychological distress. Hierarchical regression was used to estimate incremental adjusted associations rather than causal prediction. Model 1 entered demographic and art-background covariates (gender, age, grade, major type, art exposure frequency, and participation in art-related activities); Model 2 added visual art experience; Model 3 added presence of meaning and search for meaning. This order separated background characteristics from the focal exposure and then assessed whether the visual-art coefficient was attenuated after inclusion of the two meaning dimensions. The same covariates were included in the parallel indirect-effect model. Table 2 reports standardized regression coefficients, whereas the indirect-effect analysis reports unstandardized coefficients and bootstrap confidence intervals.

Regression assumptions were evaluated for the fully adjusted model using variance inflation factors (VIFs), residual distribution diagnostics, the Breusch-Pagan test for heteroscedasticity, externally studentized residuals, leverage, and Cook’s distance. Because all principal constructs were self-reported, an additional sensitivity analysis removed VAE15 (“Overall, visual art experiences have a positive influence on my psychological state”), which could overlap conceptually with the psychological-distress outcome. Cases exceeding the heuristic Cook’s-distance threshold of 4/N were also excluded in a sensitivity refit to evaluate whether influential observations changed the substantive conclusions.

To reduce reliance on the overall distress composite, the fully adjusted regression was additionally fitted for depressive symptoms, anxiety symptoms, and stress symptoms separately. Exploratory effect-modification analyses then tested whether the visual art experience-psychological distress association differed by art-related versus non-art-related major, art-activity participation, or frequency of art exposure. Major type and activity participation were tested with product terms; art-exposure frequency was treated categorically and evaluated with an omnibus three-degree-of-freedom interaction test. These moderation analyses were *post hoc* and are interpreted as exploratory. The primary parallel indirect-effect analysis used 5,000 bootstrap resamples with 95% confidence intervals; a confidence interval excluding zero was interpreted as statistical support for an indirect association, not as proof of causal mediation.

## 4 Data analysis and results

### 4.1 Data screening and sample characteristics

The analytic record included 500 submitted questionnaire records. Data-quality screening excluded 77 records: 24 for attention-check failure, 20 for completion time shorter than 180 s, 15 for five or more missing item responses, and 18 for complete straight-line responding across the major questionnaire blocks. The four archived exclusion categories were mutually exclusive, so the counts sum directly to 77. A total of 423 records were retained, corresponding to an analytic retention proportion of 84.60%. This value is not a conventional survey response rate because the number invited and the number who opened/started the survey were not retained in the project archive.

Among the 423 responses retained for analysis, 185 were male, accounting for 43.74%, and 238 were female, accounting for 56.26%. The participants ranged in age from 18 to 25 years, with a mean age of 20.34 years and a standard deviation of 1.43. In terms of grade level, 100 participants were first-year students, accounting for 23.64%; 105 were second-year students, accounting for 24.82%; 106 were third-year students, accounting for 25.06%; 74 were fourth-year students, accounting for 17.49%; and 38 were postgraduate students, accounting for 8.98%. Regarding academic major, 177 students were from art-related majors, accounting for 41.84%, while 246 students were from non-art-related majors, accounting for 58.16%.

The retained sample included students from multiple gender groups, grade levels, academic backgrounds, and levels of visual art exposure. These characteristics describe heterogeneity within the analytic sample but should not be interpreted as evidence that the sample is representative of the wider university-student population (see Table 3).

**Table 3:** Demographic characteristics of the sample.

Variable	Category	Frequency	Percentage
Gender	Male	185	43.74%
Gender	Female	238	56.26%
Grade	First-year undergraduate	100	23.64%
Grade	Second-year undergraduate	105	24.82%
Grade	Third-year undergraduate	106	25.06%
Grade	Fourth-year undergraduate	74	17.49%
Grade	Postgraduate student	38	8.98%
Major type	Art-related major	177	41.84%
Major type	Non-art-related major	246	58.16%
Frequency of visual art exposure	Almost never	63	14.89%
Frequency of visual art exposure	Once or twice per month	147	34.75%
Frequency of visual art exposure	Once or twice per week	142	33.57%
Frequency of visual art exposure	Three or more times per week	71	16.78%
Participation in art-related activities	Yes	269	63.59%
Participation in art-related activities	No	154	36.41%

*N* = 423.

### 4.2 Reliability, psychometric evaluation, and common method bias

Cronbach’s alpha was 0.902 for the visual art experience instrument, 0.780 for the adapted presence-of-meaning items, 0.754 for the adapted search-for-meaning items, and 0.912 across the 21 adapted psychological-distress items; the adapted depressive, anxiety, and stress domains had alpha coefficients of 0.850, 0.822, and 0.840, respectively. No overall alpha was calculated by combining visual art experience, meaning in life, and psychological distress because these measures represent conceptually different constructs.

For the visual art experience items, the full-sample KMO value was 0.960 and Bartlett’s test was significant (*p* < 0.001), indicating that the item correlation matrix was suitable for factor analysis. In the exploratory subsample (*n* = 211), KMO was 0.941 and Bartlett’s test was significant, χ^2^(105) = 1216.18, *p* < 0.001. Parallel analysis supported one dominant factor: the first observed eigenvalue was 6.61, exceeding the corresponding 95th-percentile random eigenvalue of 1.57, whereas the second observed eigenvalue was 0.98 and did not exceed the corresponding random eigenvalue of 1.43. Principal-axis EFA loadings ranged from 0.432 to 0.729, and the single factor accounted for 40.26% of total item variance. The one-factor CFA in the confirmatory subsample (*n* = 212) showed good fit, χ^2^(90) = 93.72, *p* = 0.373, CFI = 0.996, TLI = 0.996, RMSEA = 0.014, and SRMR = 0.039; standardized loadings ranged from 0.396 to 0.725. Composite reliability was 0.899, while AVE was 0.379. Thus, internal consistency and composite reliability were strong, but the AVE remained below the conventional 0.50 benchmark, indicating incomplete convergent validity. HTMT values between visual art experience and presence of meaning, search for meaning, depression, anxiety, and stress were 0.738, 0.153, 0.389, 0.354, and 0.339, respectively; all were below 0.85, supporting discriminant validity from these neighboring constructs. Taken together, the results support treating the instrument as a predominantly unidimensional overall score in this sample rather than as five empirically distinct subscales. Detailed split-sample loadings are reported in Supplementary Table S2. The adapted 21-item psychological-distress item set had a KMO value of 0.948, with a significant Bartlett’s test (*p* < 0.001), supporting factorability for the subsequent three-factor CFA.

For the adapted meaning-in-life items, the correlated two-factor CFA showed good fit, Chi-square (34) = 31.06, *p* = 0.613, CFI = 1.000, TLI = 1.000, RMSEA = 0.000, and SRMR = 0.034. Standardized loadings ranged from 0.595 to 0.673 for presence of meaning and from 0.570 to 0.661 for search for meaning; the estimated factor correlation was 0.123. CR was 0.780 for presence and 0.754 for search, while AVE was 0.415 and 0.381, respectively. Thus, internal consistency and composite reliability were acceptable, but AVE values below 0.50 indicate limited convergent validity. The HTMT value between presence and search was 0.132, supporting their empirical distinction. A one-factor alternative fit substantially worse, Chi-square (35) = 437.62, CFI = 0.555, TLI = 0.428, RMSEA = 0.165, and SRMR = 0.176. Item-level standardized loadings and the exact adapted wording are reported in Supplementary Table S3.

For the adapted 21-item psychological-distress measure, the correlated three-factor CFA also showed good fit, Chi-square (186) = 164.43, *p* = 0.871, CFI = 1.000, TLI = 1.000, RMSEA = 0.000, and SRMR = 0.031. Standardized loadings ranged from 0.648 to 0.696 for depressive symptoms, 0.539 to 0.688 for anxiety symptoms, and 0.618 to 0.690 for stress symptoms. Factor correlations were 0.669 between depression and anxiety, 0.737 between depression and stress, and 0.636 between anxiety and stress. CR values were 0.850, 0.823, and 0.841, and AVE values were 0.448, 0.400, and 0.430, respectively. HTMT values ranged from 0.636 to 0.741, below the 0.85 criterion, indicating empirical distinction among the three symptom domains despite their expected correlations. A one-factor alternative fit less well, Chi-square(189) = 623.30, CFI = 0.854, TLI = 0.838, RMSEA = 0.074, and SRMR = 0.068. These results support the intended three-domain organization within this sample but do not establish equivalence with the validated DASS-21. Item-level loadings are reported in Supplementary Table S4 (see Table 4).

**Table 4:** Reliability and sampling-adequacy results for conceptually distinct measures.

Scale/dimension	Number of items	Cronbach’s α	KMO	Bartlett’s χ^2^(df)	*p*
Visual art experience	15	0.902	0.960	2232.37(105)	<0.001
Presence of meaning (adapted items)	5	0.780	—	—	—
Search for meaning (adapted items)	5	0.754	—	—	—
Adapted psychological distress (21 items)	21	0.912	0.948	3133.67(210)	<0.001
Depressive symptoms (adapted items)	7	0.850	—	—	—
Anxiety symptoms (adapted items)	7	0.822	—	—	—
Stress symptoms (adapted items)	7	0.840	—	—	—

“—” indicates that KMO and Bartlett’s test are not reported for that subscale/dimension. No combined “overall scale” statistic is reported across different constructs. The meaning-in-life and psychological-distress rows refer to the study-specific adapted item sets described in Section 3.2; CFA results are reported in the text and Supplementary Tables S3, S4.

Because the focal measures were self-reported in the same survey at one time point, common method variance cannot be excluded. Harman’s single-factor test was retained only as a preliminary diagnostic: the first unrotated factor explained 23.80% of total variance. This result is not treated as evidence that common method bias is absent. Procedurally, participation was anonymous and the questionnaire used distinct response formats across construct blocks, but these features cannot eliminate common method variance. The additional VAE15-exclusion sensitivity analysis reported below was therefore used to assess whether an item with explicit psychological-state wording was disproportionately driving the focal association.

### 4.3 Descriptive statistics and correlation analysis

Descriptive statistics and Pearson correlation analysis were conducted for the main variables. The results are shown in Table 5. The mean score of visual art experience was 4.00, with a standard deviation of 0.77. The mean score of presence of meaning was 3.99, with a standard deviation of 0.87. The mean score of search for meaning was 4.00, with a standard deviation of 0.85. The mean score of psychological distress was 49.51, with a standard deviation of 25.90.

**Table 5:** Descriptive statistics and correlations among the main variables.

Variable	M	SD	1	2	3	4	5	6	7
Visual art experience	4.00	0.77	—						
Presence of meaning	3.99	0.87	0.62***	—					
Search for meaning	4.00	0.85	0.12*	0.09	—				
Depression	16.35	10.38	−0.34***	−0.37***	−0.03	—			
Anxiety	16.45	10.13	−0.31***	−0.34***	−0.06	0.56***	—		
Stress	16.70	10.11	−0.29***	−0.37***	−0.09	0.63***	0.53***	—	
Psychological distress	49.51	25.90	−0.37***	−0.43***	−0.07	0.87***	0.82***	0.85***	—

*N* = 423. *p < 0.05, **p < 0.01, ***p < 0.001.

**Table 2:** Hierarchical regression analysis of overall psychological distress.

Variable	Model 1 *β*	Model 2 *β*	Model 3 *β*
Gender, female = 1	−0.04	−0.05	−0.03
Age	0.06	0.06	0.06
Grade	0.08	0.06	0.06
Art-related major, yes = 1	0.06	0.08	0.07
Frequency of art exposure	−0.04	−0.04	−0.03
Participation in art-related activities, yes = 1	−0.06	−0.05	−0.03
Visual art experience	—	−0.37***	−0.18**
Presence of meaning	—	—	−0.31***
Search for meaning	—	—	−0.01
*R* ^2^	0.018	0.155	0.214
Adjusted *R*^2^	0.004	0.140	0.197
ΔR^2^	—	0.136	0.060
*F*	1.29	10.85***	12.53***

The dependent variable was psychological distress. Standardized regression coefficients are reported. *N* = 423. **p < 0.01, ***p < 0.001.

**Table 6:** Unstandardized bootstrap analysis of parallel indirect effects through the two meaning-in-life dimensions.

Indirect path	a path (*B*)	b path (*B*)	Direct effect c’ (*B*)	Indirect effect	Boot SE	95% bootstrap CI	Result
Visual art experience → Presence of meaning → Psychological distress	0.705	−9.285	−5.912	−6.544	1.231	[−8.988, −4.151]	Indirect effect supported
Visual art experience → Search for meaning → Psychological distress	0.137	−0.452	−5.912	−0.062	0.196	[−0.490, 0.327]	Indirect effect not supported

*B* denotes an unstandardized coefficient. The parallel model adjusts for gender, age, grade, major type, art exposure frequency, and art-activity participation. Total effect c = −12.519 (SE = 1.530, 95% CI [−15.526, −9.512]); direct effect c’ = −5.912 (SE = 1.894, 95% CI [−9.635, −2.190]).

**Table 7:** Outcome-specific adjusted regression analyses.

Outcome	VAE beta, Model 2	VAE beta, Model 3	Presence beta	Search beta	Model 3 R-squared
Depressive symptoms	−0.340***	−0.179**	−0.262***	0.018	0.170
Anxiety symptoms	−0.307***	−0.157**	−0.238***	−0.017	0.139
Stress symptoms	−0.295***	−0.108	−0.293***	−0.039	0.162
Overall psychological distress	−0.372***	−0.176**	−0.312***	−0.015	0.214

Standardized regression coefficients are shown. All models adjust for gender, age, grade, major type, art exposure frequency, and art-activity participation. Model 2 includes visual art experience; Model 3 additionally includes presence of meaning and search for meaning. **p < 0.01, ***p < 0.001.

**Table 8:** Revised summary of hypothesis testing.

Hypothesis	Statement	Result
H1	Visual art experience is negatively associated with psychological distress among university students.	Supported
H2a	Visual art experience is positively associated with presence of meaning.	Supported
H2b	Visual art experience is positively associated with search for meaning.	Supported
H3a	Presence of meaning is negatively associated with psychological distress.	Supported
H3b	Search for meaning is negatively associated with psychological distress.	Not supported
H4a	The visual art experience–psychological distress association shows a significant indirect effect through presence of meaning.	Supported
H4b	The visual art experience–psychological distress association shows a significant indirect effect through search for meaning.	Not supported

The correlation results showed that visual art experience was significantly and negatively correlated with psychological distress, *r* = −0.37, *p* < 0.001. This indicates that students with higher levels of visual art experience reported lower levels of psychological distress, providing preliminary support for H1.

Visual art experience was positively correlated with presence of meaning, *r* = 0.62, *p* < 0.001, supporting H2a. It was also positively correlated with search for meaning, *r* = 0.12, *p* < 0.05, supporting H2b, although the magnitude of this association was small.

Presence of meaning was negatively correlated with psychological distress, *r* = −0.43, *p* < 0.001, supporting H3a. By contrast, search for meaning was not significantly correlated with psychological distress, *r* = −0.07, *p* > 0.05; therefore H3b was not supported. Presence of meaning and search for meaning were themselves only weakly correlated (*r* = 0.09), further supporting their treatment as distinct dimensions rather than a single homogeneous score.

**Figure 2:**
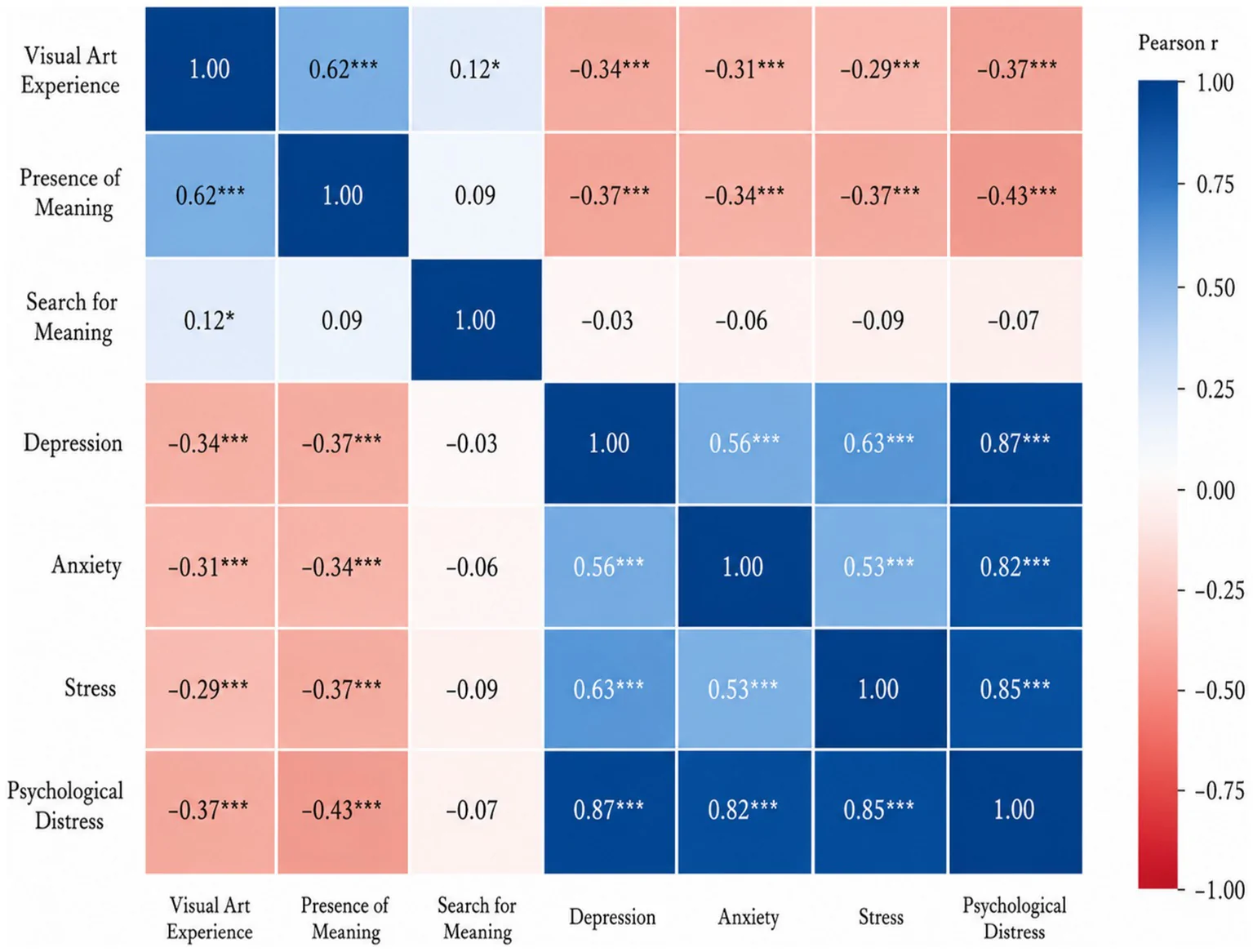
Correlation heatmap of the main variables. *** indicates *p* < 0.001.

To provide a more intuitive visualization of the correlation structure among the main variables, a correlation heatmap was generated based on the Pearson correlation coefficients reported in Table 5. As shown in Figure 2, positive correlations are displayed in blue, whereas negative correlations are displayed in red. The heatmap clearly shows that visual art experience was negatively associated with depression, anxiety, stress, and overall psychological distress. Presence of meaning also showed consistent negative associations with psychological distress and its three dimensions. In contrast, search for meaning showed relatively weak associations with psychological distress, suggesting that the two dimensions of meaning in life may play different psychological roles.

### 4.4 Regression analysis

Hierarchical regression analysis was conducted to further examine the relationships among visual art experience, meaning in life, and psychological distress. Psychological distress was entered as the dependent variable. Model 1 included only control variables, including gender, age, grade, major type, frequency of art exposure, and participation in art-related activities. Model 2 added visual art experience. Model 3 further included presence of meaning and search for meaning.

The results showed that the control variables in Model 1 explained only a small proportion of the variance in psychological distress, *R*^2^ = 0.018. This suggests that demographic characteristics and art exposure background variables alone could not sufficiently explain differences in psychological distress among university students.

After visual art experience was added in Model 2, model R^2^ increased from 0.018 to 0.155 (ΔR^2^ = 0.136). Visual art experience showed a negative adjusted association with psychological distress, *β* = −0.37, *p* < 0.001, after the demographic and art-related covariates were entered. This result is consistent with H1 but should not be interpreted as a causal effect.

In Model 3, presence of meaning and search for meaning were added separately. Model R^2^ increased to 0.214 (ΔR^2^ = 0.060). The adjusted coefficient for visual art experience was attenuated but remained significant, *β* = −0.18, *p* < 0.01. Presence of meaning was negatively associated with psychological distress, *β* = −0.31, *p* < 0.001, whereas search for meaning was not, *β* = −0.01, *p* > 0.05. This attenuation is compatible with, but does not establish, an indirect pathway through presence of meaning.

Regression diagnostics did not indicate a major violation of the linear-model assumptions. VIF values ranged from 1.02 to 1.66, indicating low multicollinearity. The fully adjusted residuals showed limited skewness (0.173) and excess kurtosis (−0.386), and the Shapiro–Wilk test was not significant (W = 0.994, *p* = 0.104). The Breusch-Pagan test did not indicate heteroscedasticity (LM chi-square = 6.11, *p* = 0.729). The maximum externally studentized residual was 2.77, with no observation exceeding an absolute value of 3. The maximum Cook’s distance was 0.021; 21 observations exceeded the heuristic 4/N threshold, but none approached 1. After excluding these 21 observations, the main pattern was unchanged (visual art experience beta = −0.208, *p* < 0.001; presence of meaning beta = −0.327, *p* < 0.001; search for meaning beta = 0.007, *p* = 0.882).

**Figure 3:**
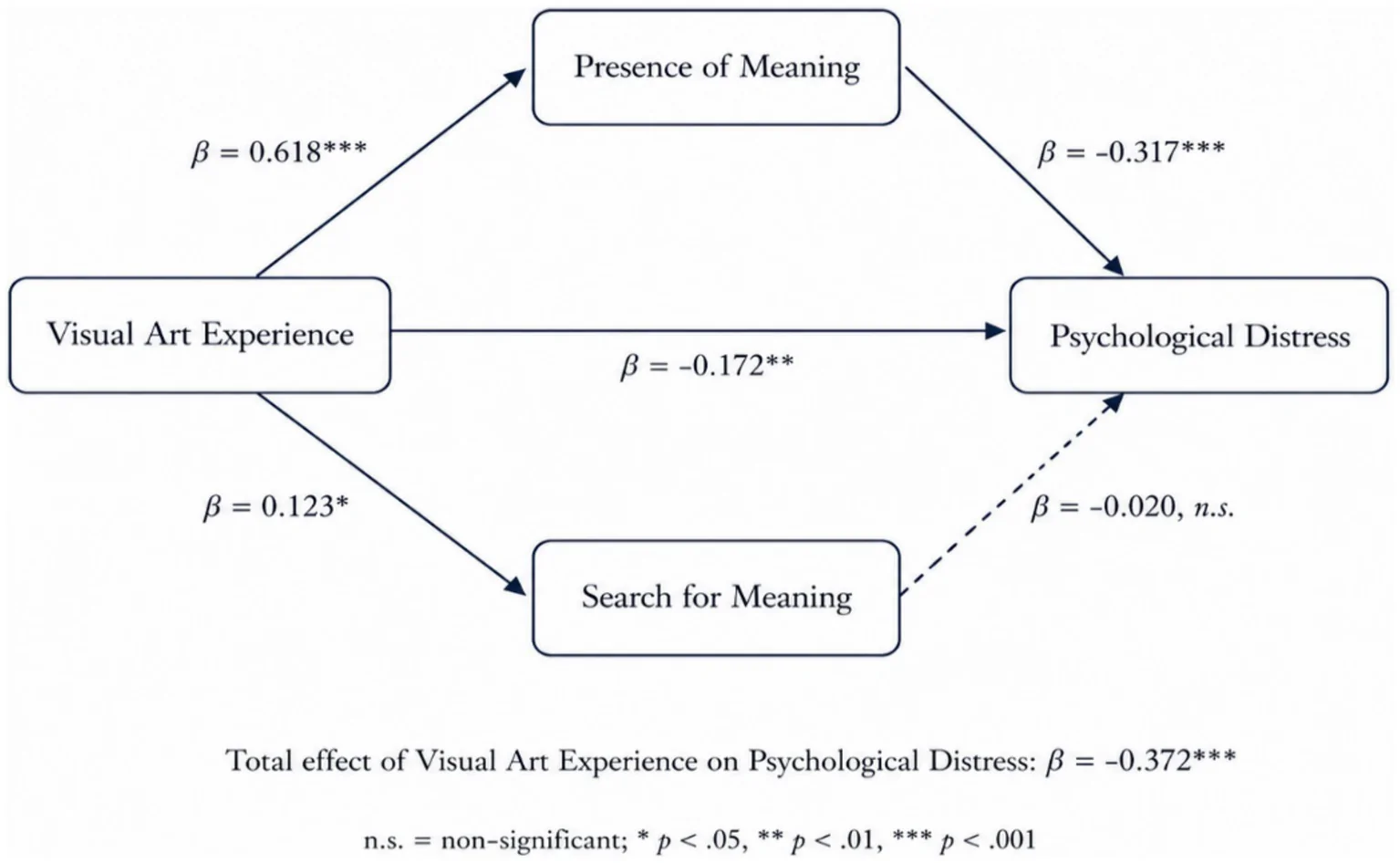
Standardized coefficients in the parallel indirect-effect model.

Figure 3 displays the standardized coefficients used to summarize the parallel model. Visual art experience was associated with both presence of meaning and search for meaning, with a substantially larger coefficient for presence of meaning. Presence of meaning was negatively associated with psychological distress, whereas the search-for-meaning path was not significant. The remaining association between visual art experience and psychological distress after inclusion of the two dimensions is consistent with a partial statistical indirect-effect pattern; causal mediation cannot be inferred.

### 4.5 Bootstrap analysis of parallel indirect effects

The primary parallel indirect-effect analysis entered presence of meaning and search for meaning simultaneously and adjusted for gender, age, grade, major type, art exposure frequency, and art-activity participation. Indirect effects were evaluated with 5,000 bootstrap resamples and 95% confidence intervals. Table 6 reports unstandardized coefficients; Figure 3 presents standardized coefficients for visual comparison. A bootstrap confidence interval excluding zero was interpreted as statistical support for an indirect association rather than evidence of a causal mechanism.

For the a paths, visual art experience was positively associated with presence of meaning (*B* = 0.705, SE = 0.044, *p* < 0.001) and search for meaning (*B* = 0.137, SE = 0.054, *p* = 0.011). In the outcome model, presence of meaning was negatively associated with psychological distress (*B* = −9.285, SE = 1.662, *p* < 0.001), whereas search for meaning was not (*B* = −0.452, SE = 1.350, *p* = 0.738). The total visual-art-experience effect was *B* = −12.519 (SE = 1.530, 95% CI [−15.526, −9.512], *p* < 0.001), and the direct effect after entering both meaning dimensions was B = −5.912 (SE = 1.894, 95% CI [−9.635, −2.190], *p* = 0.002). The indirect effect through presence of meaning was −6.544 (Boot SE = 1.231, 95% bootstrap CI [−8.988, −4.151]), whereas the indirect effect through search for meaning was −0.062 (Boot SE = 0.196, 95% bootstrap CI [−0.490, 0.327]). The two point indirect estimates sum to −6.606, corresponding descriptively to 52.8% of the total association; this proportion is not interpreted as a causal proportion mediated.

**Figure 4:**
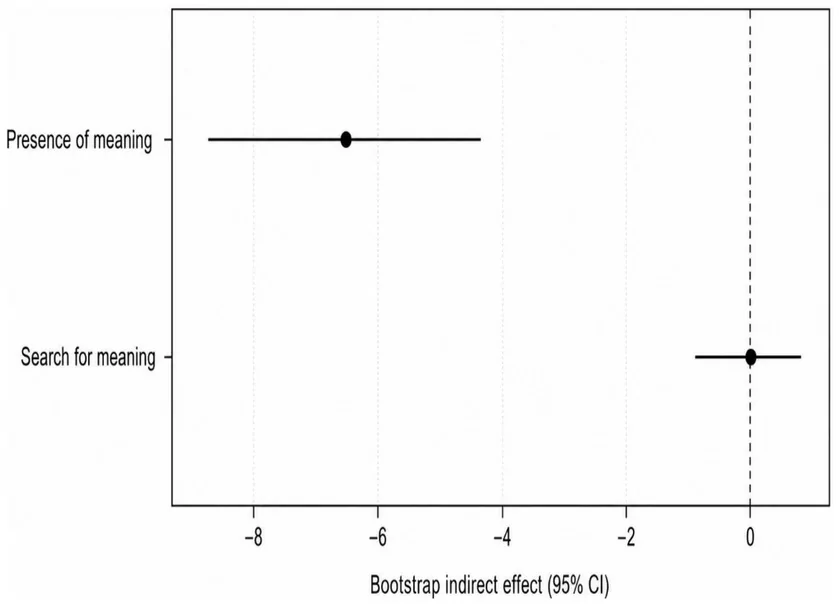
Bootstrap 95% confidence intervals for the parallel indirect effects.

The results therefore differentiate the two dimensions of meaning in life: the observed association between visual art experience and lower psychological distress was statistically consistent with an indirect path through presence of meaning, whereas the corresponding path through search for meaning was not supported (see Figure 4).

### 4.6 Domain-specific and exploratory effect-modification analyses

To examine the three psychological-distress domains directly, the hierarchical regression was repeated for depressive symptoms, anxiety symptoms, and stress symptoms using the same covariates. Before the meaning dimensions were added, visual art experience was negatively associated with all three domains (standardized beta = −0.340 for depressive symptoms, −0.307 for anxiety symptoms, and −0.295 for stress symptoms; all *p* < 0.001). After presence and search for meaning were added, the visual-art coefficient remained significant for depressive symptoms (beta = −0.179, *p* = 0.002) and anxiety symptoms (beta = −0.157, *p* = 0.008), and was attenuated for stress symptoms (beta = −0.108, *p* = 0.063). Presence of meaning remained negatively associated with all three symptom domains, whereas search for meaning was not significantly associated with any domain (see Table 7).

Exploratory interaction analyses did not provide statistical evidence that the visual art experience-psychological distress association differed by art-related major (interaction *B* = 3.455, SE = 3.051, *p* = 0.258), participation in art-related activities (interaction *B* = 2.844, SE = 3.315, *p* = 0.391), or frequency of art exposure (omnibus interaction *F*(3, 410) = 1.83, *p* = 0.141). These *post hoc* tests should not be interpreted as proof of equivalence across subgroups; rather, they indicate that effect modification was not detected in this sample.

A separate measurement-overlap sensitivity analysis excluded VAE15 because its wording explicitly referred to a positive influence on psychological state. The remaining 14-item visual art experience score retained high internal consistency (Cronbach’s alpha = 0.894) and remained negatively correlated with overall psychological distress (*r* = −0.366, *p* < 0.001). In the fully adjusted model, the 14-item score remained negatively associated with distress (beta = −0.169, *p* = 0.003), presence of meaning remained significant (beta = −0.318, *p* < 0.001), and search for meaning remained non-significant (beta = −0.015, *p* = 0.727; *R*-squared = 0.213). Thus, the principal pattern was not driven by the item with the greatest conceptual overlap with psychological state.

### 4.7 Hypothesis testing results

The hypothesis-testing results are summarized in Table 8.

## 5 Discussion

### 5.1 Visual art experience and psychological distress

Visual art experience was negatively associated with overall psychological distress and with each of the three symptom domains. The domain-specific adjusted analyses showed inverse associations with depressive, anxiety, and stress symptoms before the meaning dimensions were entered. After presence and search for meaning were added, the visual-art coefficient remained significant for depressive and anxiety symptoms and was attenuated for stress, while presence of meaning remained negatively associated with all three domains. This pattern supports reporting depression, anxiety, and stress separately rather than treating the combined distress score as the only outcome. Direct comparison with prior arts research remains cautious because formal arts education, active creation, receptive cultural participation, immersive art environments, and the broad self-reported visual art experience construct measured here are not interchangeable exposures.

One plausible interpretation is that visual art experiences provide opportunities for attentional engagement, emotional expression, symbolic processing, and reflection. These processes may coexist with lower distress, but the cross-sectional data cannot establish whether richer visual art experience precedes lower distress, whether students with lower distress engage more readily with art, or whether both are influenced by other factors. The association should therefore be viewed as preliminary and non-causal.

The present contribution is narrower than claiming a therapeutic effect of art. It identifies an association between a study-specific measure of visual art experience and self-reported psychological distress in a university sample. This result may motivate longitudinal or experimental research that distinguishes specific forms of engagement, such as creation, appreciation, immersive exposure, and formal education.

The sensitivity analysis excluding VAE15 is also important for interpretation. Because that item explicitly referred to the perceived positive influence of visual art on psychological state, it could have inflated associations through conceptual overlap with the distress outcome. Removing the item produced nearly unchanged correlations and adjusted coefficients, suggesting that the observed relationship is not attributable solely to this wording overlap. Nevertheless, all focal variables remain self-reported and common method variance cannot be eliminated statistically in a cross-sectional single-survey design.

### 5.2 Presence of meaning as a statistical indirect pathway

The parallel bootstrap analysis showed a statistically supported indirect effect through presence of meaning but not through search for meaning. This pattern is consistent with the possibility that students who report richer visual art experiences also report a stronger existing sense of purpose and coherence, which in turn is associated with lower psychological distress. The finding is described as a statistical indirect pathway because temporal ordering was not established.

The result is theoretically compatible with the MLQ distinction between presence and search (9). Presence of meaning has been consistently linked with psychological adjustment, whereas search for meaning can reflect either constructive exploration or unresolved uncertainty. The very small correlation between the two dimensions in this sample (r = 0.09) reinforces the need to interpret them separately.

Visual artworks can contain symbolic, narrative, cultural, and personally relevant material that may invite reflection on identity, relationships, memory, values, and life goals. Such processes provide a conceptual explanation for why visual art experience might be associated with presence of meaning. However, the current study did not directly measure moment-to-moment meaning-making processes, so this interpretation remains theoretical rather than demonstrated.

Research on university students indicates that meaning in life is related to self-acceptance, social support, and mental-health profiles (4, 17). The present findings add a visual-art-related correlate to this literature, while remaining agnostic about causal direction. Longitudinal designs are needed to determine whether visual art experiences contribute to later changes in meaning and distress.

From a public health education perspective, the stronger role of presence of meaning is relevant because it identifies a psychosocial resource that may be considered in future health-promotion program design and evaluation. The present data do not show that art activities cause increases in meaning, but they suggest a testable hypothesis for future longitudinal or experimental work: whether specific, well-defined art-based educational activities can strengthen an existing sense of purpose and coherence and whether such change is subsequently associated with lower distress.

### 5.3 Different roles of presence of meaning and search for meaning

A key finding is that the two dimensions of meaning in life did not show the same pattern. Presence of meaning was negatively associated with psychological distress and showed a statistically supported indirect effect. Search for meaning was only weakly related to visual art experience, was not significantly associated with psychological distress, and did not show a statistically supported indirect effect.

This difference indicates that meaning in life should not be treated as a single homogeneous construct in the present dataset. Presence of meaning reflects an existing perception of purpose and coherence, whereas search for meaning reflects an ongoing exploratory process. Combining the two dimensions into a total score could obscure these contrasting associations, which is why the revised analysis treats the parallel model as primary.

Search for meaning may have context-dependent associations: it can represent active growth-oriented exploration, but it may also coexist with uncertainty about values or life direction. The non-significant distress association observed here should therefore not be interpreted as evidence that searching for meaning is generally beneficial or harmful.

For research and educational practice, the distinction suggests that future art-based studies should measure meaning-related processes with greater precision and examine whether particular forms of art engagement are associated differently with presence and search. The present cross-sectional findings are hypothesis-generating rather than evidence that art activities produce a stable sense of meaning.

### 5.4 Implications for public health education and promotion

From a university health-promotion perspective, the present findings support further evaluation of visual-art-related educational opportunities, not the conclusion that a particular art intervention is effective. Potential future programs could be coordinated jointly by university health-promotion offices, counseling or psychological-service centers, student-affairs units, and arts/design faculty, with trained facilitators or peer leaders involved where appropriate. Activities such as visual journaling, exhibition-based reflection, photography, painting, design workshops, or digital art should be treated as examples for prospective evaluation rather than as interventions whose effectiveness has already been demonstrated by this observational study.

A tiered implementation model may be appropriate for future research. Low-threshold, optional activities could be offered universally without requiring prior art training, while more structured programs could be evaluated for students with elevated distress or low presence of meaning. Students identified as being at higher psychological risk should be assessed and referred through established campus mental-health pathways; art-based educational activities should complement rather than replace professional psychological assessment, counseling, or clinical care. The present findings therefore concern a potential educational resource within a broader system of university mental-health promotion, not a substitute treatment.

Program effectiveness should be evaluated prospectively using clearly specified exposure or intervention content, participation and reach indicators, acceptability, pre-post and follow-up measures of depressive, anxiety, and stress symptoms, presence and search for meaning, and, where feasible, comparison or randomized groups. Evaluation should also assess whether benefits and participation differ by prior art exposure, academic discipline, socioeconomic circumstances, disability status, and cultural background. The non-significant exploratory interactions in the present dataset do not establish that one program format will work equally well for all students.

Accessibility and emotional safety should be considered explicitly. Activities should minimize cost and skill barriers, provide non-performance-based options for students without formal art training, and offer accessible physical or digital formats for students with disabilities or scheduling constraints. Visual reflection can also evoke difficult memories or emotions; therefore, future programs should not be described as inherently low-risk and should include voluntary participation, clear opt-out procedures, facilitator guidance, and referral options when distress emerges.

### 5.5 Generalizability, equity, and limitations

Several limitations should be acknowledged. First, the cross-sectional design precludes temporal or causal inference; therefore, the identified indirect effects should be interpreted as statistical associations rather than evidence of a causal mechanism. Second, recruitment used a non-probability online approach within a single institutional context. Although recruitment began on April 22, 2026, the number of students who received or opened the survey link was not retained, and a probability-based sampling frame was not used. Consequently, a conventional survey response rate cannot be calculated, and the findings should not be interpreted as representative of the wider university-student population. Third, although the study received ethics approval from the Research Ethics Committee of the School of Art and Design, Ningbo University of Finance and Economics (Approval No. NBUFE-SAD-REC-2026-002; April 20, 2026), the single-institution recruitment context remains an important limitation for external validity. Future multisite studies using broader and, where feasible, probability-based recruitment strategies would help determine whether the observed associations generalize across different university populations.

Fourth, the 15-item visual art experience instrument was developed for this study. Split-sample EFA and CFA supported a predominantly unidimensional score and discriminant validity was acceptable, but AVE was below 0.50 and there was no independent pilot or external validation sample. The meaning-in-life and psychological-distress measures were also study-specific Chinese adaptations rather than verbatim administrations of the published C-MLQ or validated Simplified Chinese DASS-21. Their intended two- and three-factor structures were supported internally, but AVE values remained below 0.50 and independent translation/back-translation and external validation records were not retained. Accordingly, none of these adapted measures should be treated as fully equivalent to established validated instruments.

Fifth, all focal constructs were self-reported in the same survey, so common method variance, social desirability, and response-style effects remain possible despite the cautious Harman-test interpretation and the VAE15 sensitivity analysis. Finally, generalizability and equity require further study. The sample does not establish that visual-art-related health-promotion opportunities are equally accessible or acceptable across socioeconomic groups, disability status, cultural backgrounds, academic disciplines, or different levels of prior art exposure. Future multisite studies should deliberately sample these groups, measure barriers to participation, use accessible activity formats, and test whether effects differ across subpopulations before broad campus implementation is recommended.

Overall, the revised analyses show a consistent observational pattern: richer self-reported visual art experience was associated with lower overall psychological distress and lower depressive, anxiety, and stress symptoms, while the parallel indirect association was supported through presence of meaning but not search for meaning. Regression diagnostics, influential-case sensitivity analysis, VAE15-exclusion analysis, and exploratory subgroup interactions did not materially overturn this pattern. These findings are preliminary, cross-sectional, and hypothesis-generating; they support further longitudinal and intervention research rather than claims that visual art education itself reduces psychological distress.

## 6 Conclusion

This cross-sectional study provides preliminary observational evidence that visual art experience is associated with lower psychological distress among university students. The association was evident across depressive, anxiety, and stress symptom domains, although effect sizes were attenuated after meaning-related variables were entered. Visual art experience was positively associated with both presence of meaning and search for meaning, but only presence of meaning was negatively associated with distress and showed a statistically supported parallel indirect effect. No significant effect modification was detected by art-related major, art-exposure frequency, or participation in art-related activities in the exploratory analyses. These results identify presence of meaning as a potentially relevant psychosocial resource for future university health-promotion research, but they do not demonstrate that art-based education or any specific art activity will reduce psychological distress. Longitudinal, multisite, and experimental studies using independently validated measures and clearly specified art-based programs are needed before intervention effectiveness or causal mediation can be inferred.

## Data Availability

The original contributions presented in the study are included in the article/Supplementary material, further inquiries can be directed to the corresponding author.
