# Supplementary material for: Visual art experience and psychological distress among university students: parallel indirect effects through presence and search for meaning
Source: Front Public Health. 2026 Sep 3;14:1927579. doi: 10.3389/fpubh.2026.1927579 (PMC13582478; doi:10.3389/fpubh.2026.1927579)
Supplement: Supplementary file 1 [file Table_1.DOCX]

**Table S1. Retained Chinese wording and English rendering of the 15-item Visual Art Experience Scale**

| **Item** | **Retained Chinese wording** | **English rendering** |
| --- | --- | --- |
| VAE01 | 观看视觉艺术作品时，我常常产生情感共鸣。 | When viewing visual artworks, I often experience emotional resonance. |
| VAE02 | 视觉艺术作品能够帮助我反思自己的生活经历。 | Visual artworks help me reflect on my own life experiences. |
| VAE03 | 参与视觉艺术活动让我感到放松。 | Participating in visual art activities makes me feel relaxed. |
| VAE04 | 视觉艺术帮助我更好地理解自己和世界。 | Visual art helps me better understand myself and the world. |
| VAE05 | 接触视觉艺术时，我常常产生沉浸感。 | I often feel immersed when engaging with visual art. |
| VAE06 | 视觉艺术体验能够激发我的积极情绪。 | Visual art experiences can evoke positive emotions in me. |
| VAE07 | 视觉艺术作品会让我思考生活的价值。 | Visual artworks make me reflect on the value of life. |
| VAE08 | 我愿意主动参加视觉艺术相关活动。 | I am willing to actively participate in visual art-related activities. |
| VAE09 | 视觉艺术体验让我更容易表达自己的感受。 | Visual art experiences make it easier for me to express my feelings. |
| VAE10 | 视觉艺术作品能够带给我审美愉悦。 | Visual artworks can give me aesthetic pleasure. |
| VAE11 | 视觉艺术活动能让我暂时缓解压力。 | Visual art activities can temporarily relieve my stress. |
| VAE12 | 观看或创作视觉艺术让我感到有创造力。 | Viewing or creating visual art makes me feel creative. |
| VAE13 | 视觉艺术体验能够增强我对文化的理解。 | Visual art experiences can deepen my understanding of culture. |
| VAE14 | 我会通过视觉艺术理解他人的情绪与经历。 | I use visual art to understand other people's emotions and experiences. |
| VAE15 | 视觉艺术体验对我的日常生活具有积极意义。 | Visual art experiences have positive meaning in my daily life. |

Note. Items were scored from 1 = strongly disagree to 7 = strongly agree. The five broad content areas described in the Methods were used to organize item development conceptually and were not scored as separate subscales.

**Table S2. Split-sample factor loadings for the 15-item Visual Art Experience Scale**

| **Item** | **EFA loading (n = 211)** | **CFA standardized loading (n = 212)** |
| --- | --- | --- |
| VAE01 | 0.729 | 0.658 |
| VAE02 | 0.671 | 0.633 |
| VAE03 | 0.686 | 0.652 |
| VAE04 | 0.707 | 0.638 |
| VAE05 | 0.532 | 0.396 |
| VAE06 | 0.620 | 0.601 |
| VAE07 | 0.684 | 0.629 |
| VAE08 | 0.632 | 0.652 |
| VAE09 | 0.654 | 0.703 |
| VAE10 | 0.650 | 0.725 |
| VAE11 | 0.436 | 0.426 |
| VAE12 | 0.683 | 0.694 |
| VAE13 | 0.654 | 0.610 |
| VAE14 | 0.432 | 0.432 |
| VAE15 | 0.658 | 0.658 |

Note. The 423 retained cases were randomly split with seed = 20260810 into EFA (n = 211) and CFA (n = 212) subsamples. Parallel analysis supported one factor; principal-axis EFA loadings ranged from 0.432 to 0.729. The one-factor CFA showed chi-square(90) = 93.72, p = 0.373, CFI = 0.996, TLI = 0.996, RMSEA = 0.014, and SRMR = 0.039. Composite reliability = 0.899 and AVE = 0.379. HTMT values between VAE and presence of meaning, search for meaning, depression, anxiety, and stress were 0.738, 0.153, 0.389, 0.354, and 0.339, respectively.

**Table S3. Exact adapted meaning-in-life items and full-sample CFA standardized loadings**

| **Item** | **Dimension / Domain** | **Chinese wording** | **English rendering** | **CFA loading** |
| --- | --- | --- | --- | --- |
| MLQ01 | Presence | 我觉得自己的生活具有明确的意义。 | I feel that my life has a clear sense of meaning. | 0.673 |
| MLQ02 | Presence | 我能够理解自己生活中重要的目标。 | I can understand the important goals in my life. | 0.634 |
| MLQ03 | Presence | 我知道什么事情会让我的生活变得有价值。 | I know what makes my life valuable. | 0.664 |
| MLQ04 | Presence | 我觉得目前的生活方向较为清晰。 | I feel that the direction of my current life is relatively clear. | 0.595 |
| MLQ05 | Presence | 我能从日常经历中感受到生活的意义。 | I can experience meaning in my daily life. | 0.653 |
| MLQ06 | Search | 我经常思考自己真正想要追求什么。 | I often think about what I truly want to pursue. | 0.570 |
| MLQ07 | Search | 我仍在寻找能够使生活更有意义的目标。 | I am still searching for goals that can make my life more meaningful. | 0.653 |
| MLQ08 | Search | 我希望进一步理解自己生活的价值。 | I hope to better understand the value of my life. | 0.622 |
| MLQ09 | Search | 我会主动寻找能让我感到人生有意义的经历。 | I actively seek experiences that make my life feel meaningful. | 0.661 |
| MLQ10 | Search | 我正在努力弄清楚自己生活中最重要的事情。 | I am trying to figure out what is most important in my life. | 0.575 |

Note. Items were rated from 1 = absolutely untrue to 7 = absolutely true. The wording was adapted for this study with conceptual reference to the MLQ framework and was not a verbatim administration of the published C-MLQ. Two-factor CFA (Presence/Search): chi-square(34) = 31.06, p = 0.613, CFI = 1.000, TLI = 1.000, RMSEA = 0.000, SRMR = 0.034. CR = 0.780 (Presence) and 0.754 (Search); AVE = 0.415 and 0.381; HTMT = 0.132. The one-factor alternative showed substantially poorer fit (chi-square(35) = 437.62, CFI = 0.555, TLI = 0.428, RMSEA = 0.165, SRMR = 0.176).

**Supplementary Table S4. Exact adapted psychological-distress items and full-sample three-factor CFA standardized loadings**

| **Item** | **Dimension / Domain** | **Chinese wording** | **English rendering** | **CFA loading** |
| --- | --- | --- | --- | --- |
| PD01 | Depression | 过去一周，我对平时感兴趣的事情兴趣下降。 | During the past week, I had less interest in things I usually enjoy. | 0.649 |
| PD02 | Depression | 过去一周，我感到情绪低落或缺少愉快感。 | During the past week, I felt low in mood or lacked pleasure. | 0.648 |
| PD03 | Depression | 过去一周，我觉得很难对未来保持积极期待。 | During the past week, I found it difficult to maintain positive expectations for the future. | 0.667 |
| PD04 | Depression | 过去一周，我感觉做事情缺乏动力。 | During the past week, I lacked motivation to do things. | 0.696 |
| PD05 | Depression | 过去一周，我对自己的价值产生怀疑。 | During the past week, I doubted my own value. | 0.657 |
| PD06 | Depression | 过去一周，我觉得生活缺少活力。 | During the past week, I felt that life lacked vitality. | 0.676 |
| PD07 | Depression | 过去一周，我很难从日常活动中获得满足感。 | During the past week, I found it difficult to gain satisfaction from daily activities. | 0.690 |
| PD08 | Anxiety | 过去一周，我容易感到紧张或不安。 | During the past week, I easily felt nervous or uneasy. | 0.658 |
| PD09 | Anxiety | 过去一周，我会无明显原因地担心一些事情。 | During the past week, I worried about things without an obvious reason. | 0.622 |
| PD10 | Anxiety | 过去一周，我有时感到心跳加快或身体紧绷。 | During the past week, I sometimes felt my heart racing or my body becoming tense. | 0.688 |
| PD11 | Anxiety | 过去一周，我在压力情境下容易感到害怕。 | During the past week, I easily felt afraid in stressful situations. | 0.618 |
| PD12 | Anxiety | 过去一周，我很难让自己从担忧中平静下来。 | During the past week, I found it difficult to calm myself down from worries. | 0.539 |
| PD13 | Anxiety | 过去一周，我会担心不好的事情突然发生。 | During the past week, I worried that something bad might suddenly happen. | 0.646 |
| PD14 | Anxiety | 过去一周，我感到自己比平时更容易惊慌。 | During the past week, I felt more easily panicked than usual. | 0.649 |
| PD15 | Stress | 过去一周，我感到很难放松下来。 | During the past week, I found it difficult to relax. | 0.685 |
| PD16 | Stress | 过去一周，我容易因为小事烦躁。 | During the past week, I was easily irritated by small things. | 0.632 |
| PD17 | Stress | 过去一周，我觉得自己承受了较大的压力。 | During the past week, I felt that I was under considerable pressure. | 0.690 |
| PD18 | Stress | 过去一周，我对事情的反应比平时更急躁。 | During the past week, I reacted more impatiently than usual. | 0.640 |
| PD19 | Stress | 过去一周，我感到难以保持耐心。 | During the past week, I found it difficult to remain patient. | 0.618 |
| PD20 | Stress | 过去一周，我觉得自己一直处于紧绷状态。 | During the past week, I felt constantly tense. | 0.658 |
| PD21 | Stress | 过去一周，我很难控制自己的负面情绪反应。 | During the past week, I found it difficult to control my negative emotional reactions. | 0.665 |

Note. Every item explicitly referred to the past week and was rated from 0 = not present to 3 = very marked. The wording was adapted for this study with reference to the depression-anxiety-stress framework and was not a verbatim administration of the validated Simplified Chinese DASS-21. Three-factor CFA: chi-square(186) = 164.43, p = 0.871, CFI = 1.000, TLI = 1.000, RMSEA = 0.000, SRMR = 0.031. CR = 0.850 (Depression), 0.823 (Anxiety), and 0.841 (Stress); AVE = 0.448, 0.400, and 0.430. HTMT values were 0.672 (Depression-Anxiety), 0.741 (Depression-Stress), and 0.636 (Anxiety-Stress). The one-factor alternative fit less well (chi-square(189) = 623.30, CFI = 0.854, TLI = 0.838, RMSEA = 0.074, SRMR = 0.068). DASS-21 clinical severity cutoffs were not applied to these adapted scores.
